# Use of Simple Geometry to Predict Changes in Coronal and Sagittal Alignments Using an Extramedullary Tibial Cutting Guide During Total Knee Arthroplasty

**DOI:** 10.1016/j.artd.2020.11.005

**Published:** 2021-01-19

**Authors:** William F. Sherman, Sione A. Ofa, Travis R. Flick

**Affiliations:** Department of Orthopaedic Surgery, Tulane University School of Medicine, New Orleans, LA, USA

**Keywords:** Technique, Tibia slope alignment, Knee

## Abstract

During total knee arthroplasty, balancing is necessary for long-term stability and longevity of implants as improper balancing leads to abnormal surface strain. A routine practice among surgeons is to add more posterior slope to the proximal tibia to provide an increase in the flexion gap to balance the knee throughout the entire range of motion, particularly when doing cruciate-retaining knees. The aim of this technique guide is to provide a simple estimate of the posterior slope added or subtracted when cutting the proximal tibia using a standard extramedullary guide.  It can also be applied to predict the amount of coronal change instituted using a standard extramedullary drop guide. Using a few basic calculations with a sine equation, a surgeon can accurately predict the amount of change in the slope applied when cutting the proximal tibia. This can be done to control the degree of slope added to the anterior-posterior direction and can be used to predict coronal alignment changes as well. This technique can be applied to any length extramedullary guide and applied across all companies to provide surgeons with an exact degree change in the tibial slope and coronal alignment with simple calculations.

## Introduction

Total joint arthroplasty is among the most successful orthopedic surgeries currently performed, with greater than 90% long-term survivorship at 15 years for total knee arthroplasty (TKA) [1,2]. During TKA, balancing is necessary for long-term stability and longevity of implants as improper balancing leads to abnormal surface strain [3]. With differences in balancing techniques including gap balancing, measured resection, and kinematic alignment, surgeons often make alignment changes from the initial surgical cuts to account for asymmetric gaps.

The mean tibial posterior slope in the medial plateau has been documented to be between 6.8 and 10.7 degrees and the mean tibial posterior slope in the lateral plateau between 7.2 and 8.0 degrees in the normal knee [4,5]. The addition of the tibial slope during TKA to provide a larger flexion gap during knee balancing is commonly done when there is asymmetric tightness in flexion and the surgeon either has already moved the femoral component anteriorly or prefers to keep the femoral component in place and adjust the tibial slope to balance the flexion gap [6]. Other surgical options for increasing the flexion gap are to sacrifice or recess the posterior cruciate ligament (PCL) [7]. Because the posterior slope can also act in concert sharing the role of the posterior cruciate ligament in preventing posterior translation of the tibia during loading, the addition of the slope is often performed when recession of the PCL is needed [8].

Several methods to achieve a desired coronal and sagittal alignment are currently in use including computer navigation, intramedullary tibial guides, extramedullary tibial guides, and robot-assisted arms [9,10]. The aim of this technique guide is to provide a simple estimate of the joint line changes with distal adjustment of a tibial guide rod when cutting the proximal tibia using standard extramedullary instrumentation controlling for the length of the guide rod. This method can also be applied to predict the amount of coronal change using a standard extramedullary guide rod.

## Surgical technique

To obtain a measurement for the posterior slope, a line drawn perpendicular to the long axis of the tibia was defined as the reference line and represented 0^o^ slope. A line was then drawn parallel to the articular surface of the proximal tibia. The angle between the reference line and the articular surface line was designated as the tibial slope angle. If the slope proceeded from anterior-superior to posterior-inferior, it was designated a positive value. If the posterior tibia had a higher elevation than the anterior tibia, then the slope was called an anterior slope and was given a negative value.

**Figure 1:**
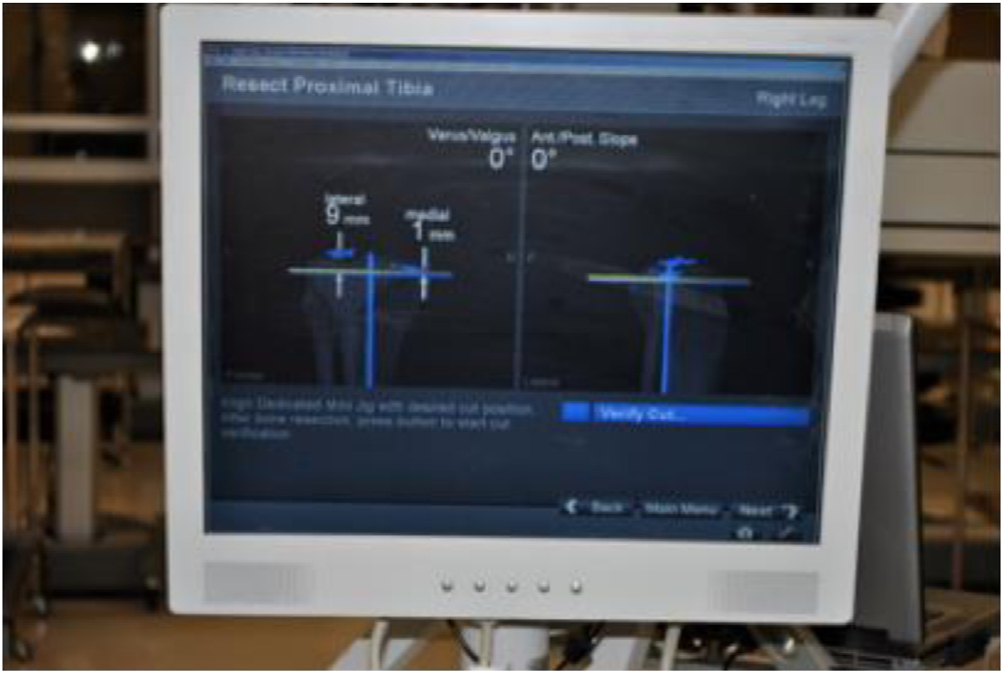
Navigation demonstrating measurements.

After measuring the length of the extramedullary tibial guide rod, the distance from the proximal tibia at the level of resection, and the distance of the drop guide to the anterior tibia, the addition of slope can be precisely measured using the following formula: Sin (plateau slope) = opposite/hypotenuse. This same principle can be applied to the amount of coronal angulation added in reference to the amount of medialization and lateralization of the distal end of the drop guide. The geometry was measured, predicted, and confirmed on a cadaver using navigation to demonstrate all measurements (Fig. 1).

**Figure 2:**
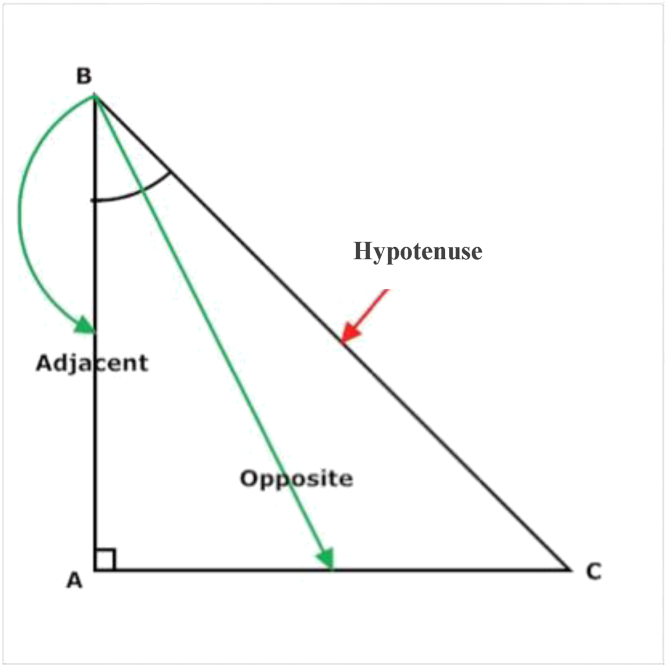
Right triangle for visualization.

**Figure 3:**
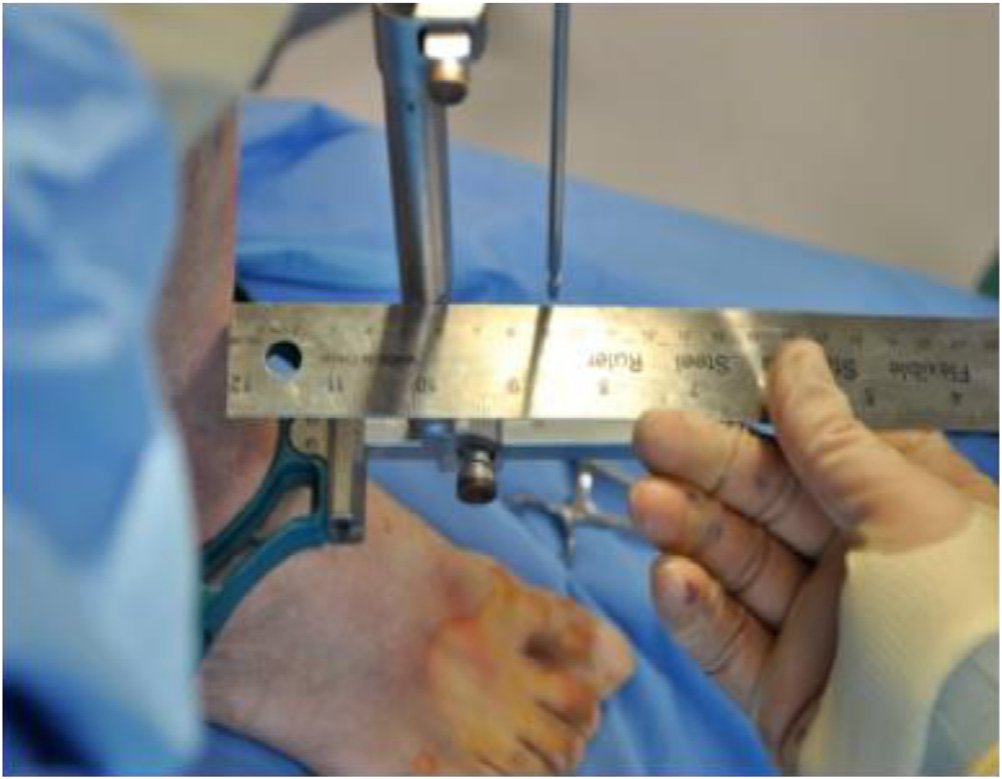
Measuring opposite distance to predict the slope.

Using a right triangle (Fig. 2), the distance (hypotenuse) was substituted for with 25 cm to represent an extramedullary guide rod. At this point, any distance added as the opposite can accurately predict the amount of slope (represented by B) added to an extramedullary guide. Using a metal ruler (Fig. 3) to measure and mark distance of the opposite, we are able to accurately predict the amount of the posterior slope added depicted as B.

**Figure 4:**
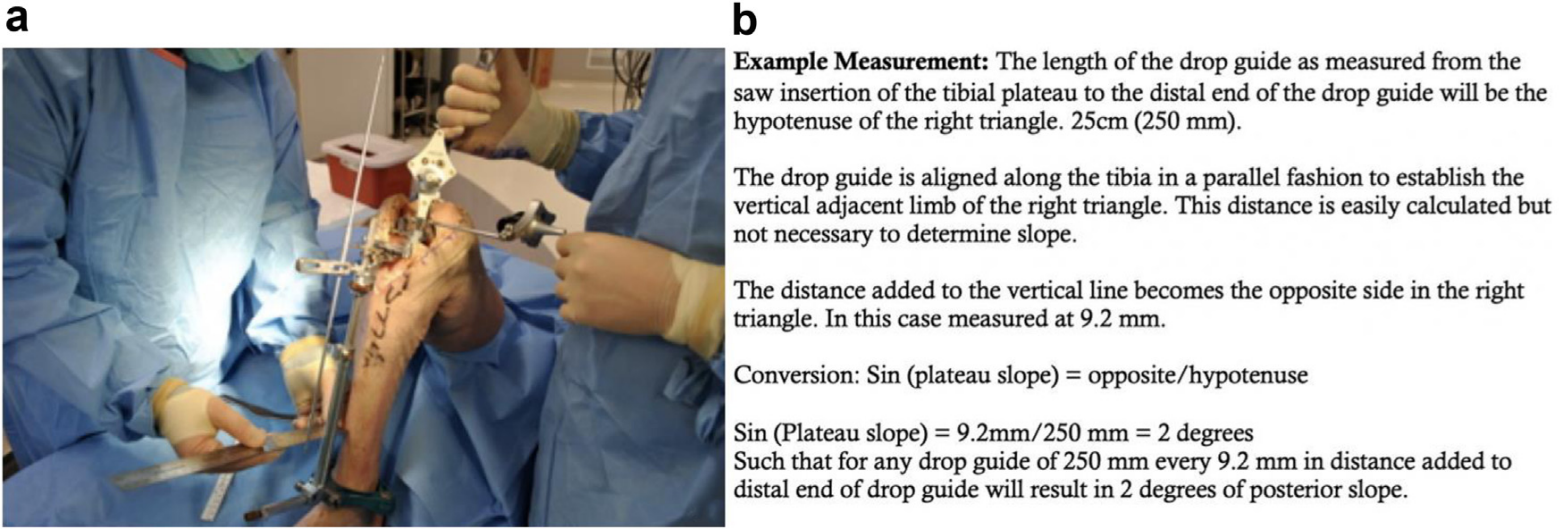
(a) Surgeon demonstrating measurements on a cadaver. (b) Example of measurements being calculated.

This flow can be made simple by understanding that the length of the drop guide will influence changes in the alignment at the joint line. In the shorter tibia, the distal adjustments will have greater joint line changes, whereas on the longer tibia, the majority of extramedullary guides will be extended in length such that the distal adjustments have less effect on joint line changes. W. Sherman (Fig. 4) references a set point on the extramedullary guide at 25 cm of length and knows that when changing the slope or coronal alignment, one fingerbreadth of distal movement of the guide rod translates to 2 degrees of alignment change at the joint line assuming one fingerbreadth is 1 cm. For patients with increased tibial soft tissue and a high body mass index, the tibial guide rod is often hard to directly align with the tibia in the anterior-posterior plane. This knowledge can allow a surgeon to know if the guide rod is indeed displaced distally, one can expect 2 degrees of added slope for each fingerbreadth distally that the guide is moved away from the tibia at the 25-cm mark on an extramedullary guide rod.

## Discussion

There are many decisions made in regard to balancing during primary TKA that include ligament, tendon, and bony resections. Oftentimes, these small adjustments are made in concert to achieve a balanced knee throughout the range of motion. One of the most common intraoperative alignment decisions surgeons make is the amount of posterior slope chosen for a specific patient. Each implant is designed for an optimum slope taking into account the radius of curvature of the femur and the design of the polyethylene. With several methods of balancing that can involve both soft-tissue releases and bony cuts to achieve a balanced knee, the surgeon has to choose what combinations to use to achieve this balance while taking into account coronal and sagittal alignment changes that will result from these alterations. Knowledge of how these small changes affect the mechanical alignment is critical for a surgeon to reach an optimal outcome.

## Summary

Regardless of the type and length of the drop guide currently used in a system during TKA, a surgeon can measure the guide-rod length before surgery, and with a few basic calculations using a sine equation, can accurately predict the amount of change in the slope applied when cutting the proximal tibia. This can be performed to control the degree of slope added to the anterior-posterior direction and can be used to predict coronal changes as the same distance changes at the tip of the guide rod at a constant length are linear with the degree change.

## Conflicts of Interest

The authors declare there are no conflicts of interest.

## Supplementary data

Conflict of Interest Statement for Ofa

Conflict of Interest Statement for Flick

Conflict of Interest Statement for Sherman

## References

[1] Jauregui J.J., Cherian J.J., Pierce T.P., Beaver W.B., Issa K., Mont M.A. (2015). Long-term survivorship and clinical outcomes following total knee arthroplasty. J Arthroplasty.

[2] Vessely M.B., Whaley A.L., Harmsen W.S., Schleck C.D., Berry D.J. (2006). The Chitranjan Ranawat Award: long-term survivorship and failure modes of 1000 cemented condylar total knee arthroplasties. Clin Orthop Relat Res.

[3] Green G.V., Berend K.R., Berend M.E., Glisson R.R., Vail T.P. (2002). The effects of varus tibial alignment on proximal tibial surface strain in total knee arthroplasty: the posteromedial hot spot. J Arthroplasty.

[4] Matsuda S., Miura H., Nagamine R. (1999). Posterior tibial slope in the normal and varus knee. Am J Knee Surg.

[5] Nunley R.M., Nam D., Johnson S.R., Barnes C.L. (2014). Extreme variability in posterior slope of the proximal tibia: measurements on 2395 CT scans of patients undergoing UKA?. J Arthroplasty.

[6] Lombardi A.V., Berend K.R., Aziz-Jacobo J., Davis M.B. (2008). Balancing the flexion gap: relationship between tibial slope and posterior cruciate ligament release and correlation with range of motion. J Bone Joint Surg Am.

[7] Scott R.D., Chmell M.J. (2008). Balancing the posterior cruciate ligament during cruciate-retaining fixed and mobile-bearing total knee arthroplasty: description of the pull-out lift-off and slide-back tests. J Arthroplasty.

[8] Whiteside L.A., Amador D.D. (1988). The effect of posterior tibial slope on knee stability after Ortholoc total knee arthroplasty. J Arthroplasty.

[9] Gaudiani M.A., Nwachukwu B.U., Baviskar J.V., Sharma M., Ranawat A.S. (2017). Optimization of sagittal and coronal planes with robotic-assisted unicompartmental knee arthroplasty. Knee.

[10] Nam D., Cody E.A., Nguyen J.T., Figgie M.P., Mayman D.J. (2014). Extramedullary guides versus portable, accelerometer-based navigation for tibial alignment in total knee arthroplasty: a randomized, controlled trial: winner of the 2013 HAP Paul award. J Arthroplasty.

