# Supplementary material for: Use of Simple Geometry to Predict Changes in Coronal and Sagittal Alignments Using an Extramedullary Tibial Cutting Guide During Total Knee Arthroplasty
Source: Arthroplast Today. 2021 Jan 19;7:250–251.e1. doi: 10.1016/j.artd.2020.11.005 (PMC7987928; doi:10.1016/j.artd.2020.11.005)
Supplement: Conflict of Interest Statement for Sherman [file mmc3.docx]

# INDIVIDUAL CONFLICT OF INTEREST STATEMENT

***American Association of Hip and Knee Surgeons***

(Adopted from the American Academy of Orthopaedic Surgeons disclosure statement)

The following form **must be filled out completely and submitted by each author (example, 6 authors, 6 forms).**

**All items require a response. If there is no relevant disclosure for a given item, enter "*None*.”**

**Manuscript Title:** Use of simple geometry to predict changes in coronal and sagittal alignment using an extra-medullary tibial cutting guide during Total Knee Arthroplasty (TKA)

1. Royalties from a company or supplier (The following conflicts were disclosed)

none

2. Speakers bureau/paid presentations for a company or supplier (The following conflicts were disclosed)

none

3A. Paid employee for a company or supplier (The following conflicts were disclosed)

none

3B. Paid consultant for a company or supplier (The following conflicts were disclosed)

none

3C. Unpaid consultants for a company or supplier (The following conflicts were disclosed)

none

4. Stock or stock options in a company or supplier (The following conflicts were disclosed)

none

5. Research support from a company or supplier as a Principal Investigator (The following conflicts were disclosed)

none

6. Other financial or material support from a company or supplier (The following conflicts were disclosed)

none

7. Royalties, financial or material support from publishers (The following conflicts were disclosed)

none

8. Medical/Orthopaedic publications editorial/governing board (The following conflicts were disclosed)

none

9. Board member/committee appointments for a society (The following conflicts were disclosed)

none

**Each author must sign AND print or type his/her name, date and submit a separate form**

In addition, one BLINDED Conflict of Interest form (no author names used) should be submitted per manuscript with all author disclosures.

WIlliam Franklin Sherman 6/9/2020

Author Name (Print or Type) Author Signature Date
